# Real-World Effectiveness and Safety of Golimumab in Patients with Ulcerative Colitis: A Retrospective Cohort Study

**DOI:** 10.3390/jcm14196827

**Published:** 2025-09-26

**Authors:** Rodrigo Galhardi Gasparini, Carlos Taxonera, Antônio José Tibúrcio Alves Júnior, Bianca Loyo Pona Schiavetti, Francisco Guilherme Cancela e Penna, Richard Borba Magalhães, Sandro da Costa Ferreira, Renata de Sá Brito Fróes, Carlos Henrique Marques dos Santos, Cristina Flores, Bruno César da Silva, Rogério Serafim Parra, Eduardo Federighi Baisi Chagas, Alexandre Venâncio, Hélio Rzetelna, Carlos Frederico Pereira Porto Alegre Rosa, Adriana Ribas Andrade, Caio Cesar Furtado Freire, Mikaell Alexandre Gouvea Faria

**Affiliations:** 1 Sete Specialized Medical Center, Marília 17.502-020, Brazil; 2 IBD Unit, Hospital Universitario Clínico San Carlos, 28040 Madrid, Spain; 3 Medical School, Pontifícia Universidade Católica de Campinas, Campinas 13.086-105, Brazil; antonio_xxxv@yahoo.com.br; 4 Santos City Hall—Santa Casa de Santos, Santos 11.010-080, Brazil; biancalps@hotmail.com; 5 Hospital das Clínicas da Universidade Federal de Minas Gerais, Belo Horizonte 30.130-100, Brazil; cancelapenna@gmail.com; 6 Centro de Doenças Inflamatórias Intestinais e Imunomediadas, Porto Alegre 90.480-000, Brazil; richardbm@me.com (R.B.M.); cfloresgastro@gmail.com (C.F.); 7 Ribeirão Preto Medical School, University of São Paulo, Ribeirão Preto 14.048-900, Brazil; scferreira@hcrp.usp.br (S.d.C.F.); rsparra@hcrp.usp.br (R.S.P.); 8 Gastomed, Rio de Janeiro 22.640-100, Brazil; refroes@gmail.com; 9 Hospital Regional de Mato Grosso do Sul, Campo Grande 79.084-180, Brazil; chenriquems@yahoo.com.br; 10 Hospital da Bahia, Salvador 41.810-011, Brazil; bcesars@hotmail.com; 11 Postgraduate Program, Universidade de Marília, Marília 17.525-902, Brazil; efbchagas@gmail.com; 12 Faculdade de Medicina de Jundiaí, Jundiaí 13.202-550, Brazil; medicina13@yahoo.com.br; 13 Quinta D’or Hospital, Rio de Janeiro 20.941-150, Brazil; hrzetelna@gmail.com; 14 Oeste D’or Hospital, Rio de Janeiro 23.045-160, Brazil; fredpa@gmail.com; 15 Department of Life Science, Bahia State University, Salvador 41.150-000, Brazil; adriana.ribas.andrade@gmail.com; 16 Intestino Center, Fortaleza 60.135-101, Brazil; caiofreire@yahoo.com.br; 17 Clinica Flamini, São José do Rio Preto 15.091-020, Brazil

**Keywords:** inflammatory bowel disease, ulcerative colitis, golimumab, Brazil

## Abstract

**Background/Objectives:** Golimumab has proven efficacy in inducing and maintaining remission in moderate-to-severe ulcerative colitis (UC). This study evaluated the short-term and long-term effectiveness and safety of golimumab for the treatment of patients with active UC in Brazil. **Methods:** This observational, multicenter, retrospective, cohort study included patients with moderate-to-severe UC treated with golimumab. The primary outcome was corticosteroids-free clinical remission at weeks 24 and 54, defined as a partial Mayo score (PMS) of 0 or 1, without the need for corticosteroids (CS,) in continued treatment with golimumab. Secondary outcomes were clinical response, defined as a reduction in PMS of 50% or 3 points, and endoscopic remission, defined as a Mayo endoscopic subscore of 0. We also evaluated persistence with golimumab during follow-up. **Results:** Seventy-three patients were enrolled in the study. The rates of CS-free remission at weeks 24 and 54 were 43.8% and 63%, respectively. Clinical response was achieved in 50.7% and 71.2% of patients at weeks 24 and 48, respectively. Among patients undergoing endoscopic evaluation, CS-free endoscopic remission was observed in 80.8% of patients at week 24 and in 84.4% at week 54. The cumulative probability of retaining golimumab was 86.1% (95% CI 78–94) at 54 weeks. Adverse events leading to golimumab discontinuation occurred in three patients (4.1%). **Conclusions:** Golimumab was effective and safe as induction and maintenance therapy in patients with moderate-to-severe ulcerative colitis, leading to a high rate of persistence with golimumab maintenance after 1 year of follow-up.

## 1. Introduction

Ulcerative colitis (UC) is a chronic inflammatory condition of the colon, that manifests as bloody diarrhea, weight loss, and abdominal pain. The disease has a recurring course and requires lifelong medical management. Historically, corticosteroids (CS), 5-aminosalicylates (5-ASA), and thiopurines have been the primary therapeutic agents for UC. However, biological medications and, more recently, Janus kinase (JAK) inhibitors, have emerged as the preferred treatment options for patients with moderate-to-severe disease who exhibit inadequate response or intolerance to conventional therapies [1,2,3,4]. Although JAK inhibitors represent a more recent advance with promising efficacy, it was the introduction of antibodies to tumor necrosis factor alpha (TNF-α) that truly transformed the therapeutic landscape, drastically changing the management of moderate-to-severe UC by enabling mucosal healing in a significant number of patients and considerably reducing colectomy and hospitalization rates [5,6,7]. Currently, three anti-TNF-α agents are approved for the treatment of UC: infliximab, adalimumab, and, more recently, golimumab. Golimumab is a fully human IgG1 kappa monoclonal antibody that targets both membrane-bound and soluble TNF-α. Developed through transgenic human antibodies, it offers clinical benefits such as higher affinity and binding power to TNF-α, achieving therapeutic goals with less drug and fewer side effects [8]. Its stability allows subcutaneous (SC) administration without citrate, thereby reducing injection site reactions and pain. High solubility minimizes biological molecule aggregation, thus lowering immunogenicity and the formation of anti-drug antibodies. These features support a 4-week SC dosing regimen with low injection volumes.

Golimumab is used in clinical practice as an induction and maintenance agent in the treatment of UC. The efficacy and safety of golimumab in treating moderate-to-severe UC were demonstrated in the induction and maintenance randomized clinical trials (RCTs) of the PURSUIT (Ulcerative Colitis Research Study Program Using an Investigational Treatment) program [9,10]. However, the strict inclusion criteria used in RCTs can limit the patient population and generalizability of trial results to clinical practice. Therefore, it is important to understand the effectiveness and safety of this treatment in the real-world clinical setting and in different patient populations. Few observational studies have been reported on the long-term outcomes of golimumab for UC. These studies have shown highly variable golimumab therapy retention in some cohorts and reduced long-term persistence in anti-TNF-α multi-experienced patients [11,12,13,14,15]. There are currently no real-world data on the use of golimumab in patients with UC in Brazil. The objective of this observational study was to evaluate the effectiveness and safety of golimumab for the treatment of patients with active UC in Brazil. As secondary objective we explored the predictors of effectiveness outcomes, including persistence with golimumab.

## 2. Materials and Methods

### 2.1. Study Design and Patients

This was a multicenter, observational, retrospective, cohort study of patients with UC treated with golimumab. This study was conducted and reported according to the methods and recommendations from the STrengthening the Reporting of OBservational studies in Epidemiology (STROBE) [16]. The study was approved by the FAMEMA Research Ethics Committee (CEP) on 28 June 2023, under protocol nº 69134923.8.1001.5413.

Eligible patients were men or women of at least 18 years of age with an established diagnosis of UC according to Lennard–Jones criteria [17]. The study population comprised all consecutive patients with moderate-to-severe active UC who received golimumab induction doses and had at least 24 weeks of follow-up. Golimumab was administered according to the dosage approved for UC in the summary of product characteristics by the Brazilian Health Regulatory Agency (ANVISA). Golimumab was given SC as an initial dose of 200 mg, followed by 100 mg at week 2. Patients who responded to golimumab induction and had a body weight of less than 80 kg or with body weight greater than or equal to 80 kg received golimumab maintenance doses of 50 or 100 mg every 4 weeks (q4wk), respectively. Escalation of golimumab during maintenance (by increasing the dose or shortening the interval) was at the discretion of the treating physician. These optimizations included doses that exceeded ANVISA approval but were in accordance with clinical practice [18,19].

### 2.2. Baseline Assessments

Baseline clinical data and information on therapy outcomes were collected through a retrospective review of medical records. Baseline variables collected included age, age at UC diagnosis, weight, height, body mass index (BMI), sex, ethnicity, smoking status, comorbidities, extraintestinal manifestation (EIM), date of symptom onset, disease duration, extent of colitis, use of 5-ASA, use of immunomodulator (thiopurines/methotrexate), previous exposure to biological therapy (infliximab, adalimumab, certolizumab, vedolizumab, and ustekinumab) and use of CS. Baseline disease activity was evaluated using the Mayo score (0–12), the partial Mayo score (PMS; 0–9), and levels of C-reactive protein (CRP) and fecal calprotectin. When baseline endoscopic examinations were available, the Mayo endoscopic subscore (MES; 0–3) was assessed [20]. The golimumab maintenance regimen was documented, including the need for dose escalation.

### 2.3. Study Endpoints

The primary outcome was CS-free clinical remission at weeks 24 and 54, defined as a PMS of 0 or 1, without the need for CS, in patients receiving continued treatment with golimumab. Secondary outcomes included clinical response, defined as a reduction in the PMS of 50% or 3 points, and endoscopic remission, defined as an MES of 0, in continued treatment with golimumab. Rates of clinical response, CS-free clinical remission, and CS-free endoscopic remission were assessed at weeks 8, 24, 36, and 54 were available. Persistence with golimumab, i.e., the period during which patients remained free from discontinuation of the drug during follow-up, was also evaluated. Other secondary outcomes included golimumab dose optimization and the time and reason for golimumab discontinuation. Golimumab discontinuation due to primary failure was defined as discontinuation of the drug due to lack of response within three months of starting therapy. Secondary failure was defined as drug discontinuation due to lack of response at least three months after initiating golimumab. Patients who had secondary loss of response but subsequently regained response following golimumab dosage optimization were not considered as failures. For patients who continued golimumab treatment, therapy time was measured from the start of therapy until the last follow-up in weeks. For patients who discontinued golimumab, therapy time was measured from the initiation of golimumab until discontinuation in weeks. The time to golimumab dose escalation was defined as the number of weeks from the start of therapy until the dose was increased or the dosing interval was reduced. Dose escalation before week 12 was defined as early optimization. We also evaluated the variation of clinical outcomes and of CRP and fecal calprotectin levels between baseline and the different time points. We analyzed predictors of clinical response or remission at weeks 24 and 54. The model included variables that evaluated the impact of demographic factors (sex, age), disease-related characteristics (duration and extent of ulcerative colitis, according to the Montreal classification), treatment-related variables (corticosteroid use at baseline, concomitant immunomodulator therapy, and prior exposure to biologics), and smoking status. The rate of adverse events was also analyzed.

### 2.4. Statistical Analysis

Qualitative variables are described by the absolute and relative frequency distribution (%). The difference in frequency distribution of qualitative variables was analyzed using the chi-square proportion test. Quantitative variables were described as the mean and standard deviation (SD), or median and interquartile range (IQR) (P25-P75). The 95% confidence interval (CI) was calculated using the bootstrap technique for percentiles, considering a resampling of 1000 sample elements. In the long-term, golimumab failure-free survival and golimumab dose optimization-free survival rates were estimated using survival analysis. The cumulative probability of the event-free survival was calculated by the Kaplan–Meier method. To investigate factors potentially associated with golimumab effectiveness, we conducted both univariable and multivariable Cox proportional hazards regression analyses. In the univariable analysis, each predictor was individually tested to assess its association with the outcomes of interest at predefined timepoints. Variables with a *p*-value < 0.10 in the univariable models, or those deemed clinically relevant based on existing literature (e.g., disease extent, immunosuppressant use, biologic exposure), were subsequently included in the multivariable analysis, which adjusted for potential confounding and identified independent predictors.

For repeated measures of clinical outcomes, the repeated measures ANOVA or Greenhouse–Geisser correction test were performed when data violated the sphericity assumption by Mauchly’s test. Post hoc comparisons were conducted using the Bonferroni test. To compare the median between weeks, the Friedman test was used, followed by the Holm–Sidak post hoc test. The association between qualitative variables was analyzed using the chi-square test, as well as the analysis of relative risk (RR) for variables with dichotomous outcomes. The adopted significance level was 5%, and the data were analyzed using Jamovi software (version 2.6.26) and SPSS software (version 27.0).

## 3. Results

### 3.1. Baseline Demographics

A total of 73 consecutive patients with active UC who were treated with golimumab at fourteen medical centers across Brazil were included in the study. The patients’ baseline characteristics are summarized in Table 1, Table 2 and Table 3. All patients had moderate-to-severe active UC, and 63 patients (86.3%) were using CS at baseline. At baseline, 55 patients (75.4%) weighed < 80 kg and received a golimumab maintenance dose of 50 mg q4wk; the remaining 18 patients (24.6%) weighed ≥80 kg and received 100 mg q4wk. Twenty-six patients (35.6%) had previously been exposed to thiopurines, and 24 patients (32.9%) had previously been exposed to biologics: nine (13.3%) received infliximab, five (6.8%) adalimumab, thirteen (17.8%) vedolizumab, three (4.1%) certolizumab pegol, and one (1.4%) ustekinumab; of these, one (1.4%) patient was exposed to both adalimumab and vedolizumab, one (1.4%) to infliximab and adalimumab, one (1.4%) to certolizumab and adalimumab, two (2.7%) to vedolizumab and infliximab, and two (2.7%) to adalimumab, infliximab and vedolizumab.

### 3.2. Persistence on Golimumab Therapy

**Figure 1:**
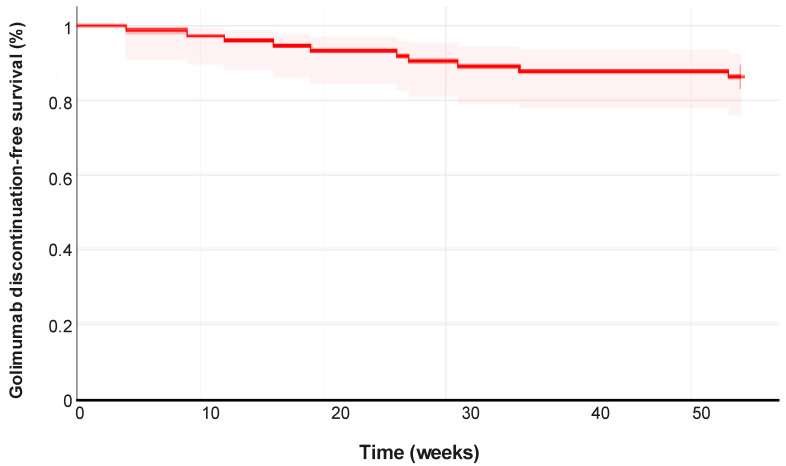
Cumulative golimumab discontinuation-free survival in the study cohort (*n* = 73). Ten patients discontinued golimumab during follow-up. The median time to golimumab discontinuation was 25.3 weeks (IQR 30).

The mean duration of golimumab therapy was 59.8 weeks (95% CI 49.5–70.0). The cumulative probability of retaining golimumab was 86.1% (95% CI 78–94) at 54 weeks (Figure 1). Ten patients (13.7%, 95% CI 06–22) discontinued golimumab during follow-up, comprising seven men (70%) and three women (30%). Median time to golimumab discontinuation was 25.3 weeks (IQR 30.5, range 4–55). The reasons for discontinuation were primary failure in two patients (2.73%), secondary failure in two patients (2.73%), and other causes in six patients (8.2%). Among the four patients who discontinued golimumab due to failure, two (2.73%) were anti-TNF-experienced, and two (2.73%) had experience with vedolizumab.

### 3.3. Primary and Secondary Outcomes

**Figure 2:**
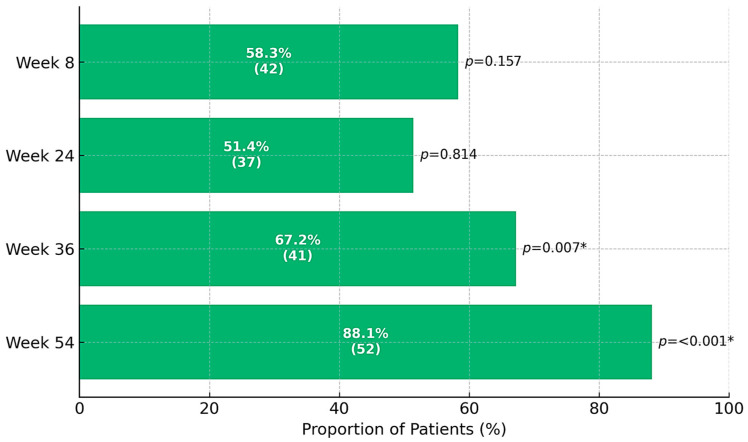
Absolute (*n*) and relative (%) frequency distribution for clinical response during follow-up (*n* = 73).* indicates a significant difference in the proportion distribution between the categories for % valid by the chi-square test for *p*-value ≤ 0.050.

**Figure 3:**
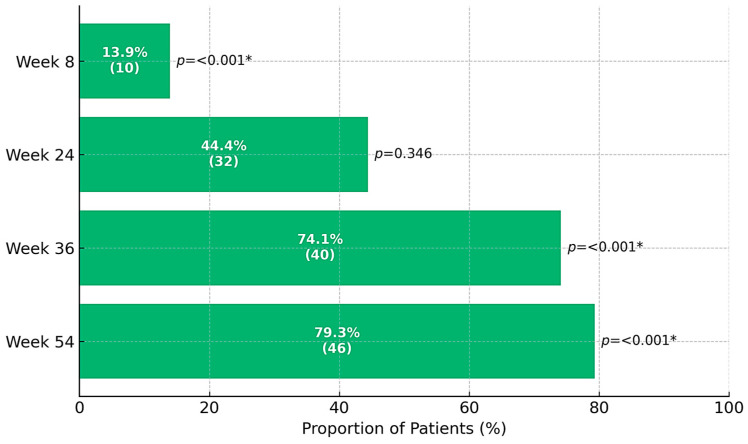
Absolute (*n*) and relative (%) frequency distribution for corticosteroid-free (CS-free) clinical remission during follow-up (*n* = 73). * indicates significant difference in the distribution of proportions between categories for % valid by the chi-square test for *p*-value ≤ 0.050.

**Figure 4:**
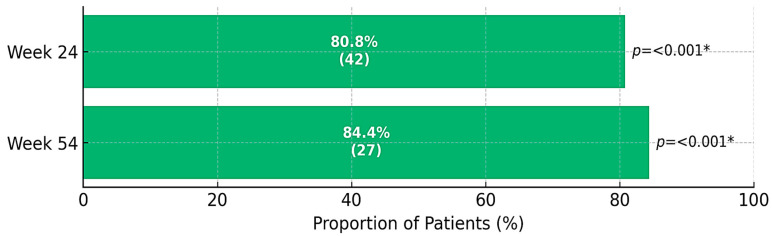
Absolute (*n*) and relative (%) frequency distribution for corticosteroid-free (CS-free) endoscopic remission during follow-up (*n* = 73). * indicates significant difference in the distribution of proportions between categories for % valid by the chi-square test for *p*-value ≤ 0.050.

Rates of clinical response, CS-free clinical remission, and CS-free endoscopic remission at the follow-up time points are presented in Figure 2, Figure 3 and Figure 4. The primary endpoint, CS-free clinical remission, was achieved in 32 of 73 patients (43.8%, 95% CI 41.5–67.9) at week 24, and in 46 of 73 patients (63%, 95% CI 44.4–88.9) at week 54. Thirty-seven patients (50.7%) and 52 patients (71.2%) had clinical response at weeks 24 and 54, respectively. Four patients (5.47%) needed colectomy due to medical refractoriness after a median time of 28 weeks (IQR 40.5, range 11–92), three (75%) of them had previously been exposed to biologics. Sixty-three patients (86.3%) were using CS at baseline: 45 of these (71.4%) were able to withdraw from CS at week 24. At least one follow-up endoscopy was performed in 56 patients (76.7%) after a median of 24 weeks (IQR 24, range 4–60) after starting golimumab. Among patients undergoing endoscopic evaluation, CS-free endoscopic remission was observed in 42 of 52 patients (80.8%) at week 24 and 27 of 32 patients (84.4%) at week 54.

**Figure 5:**
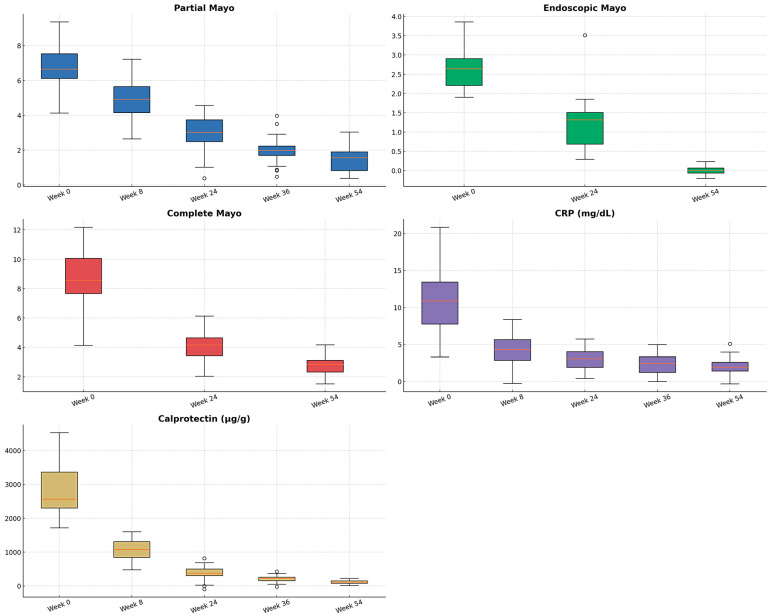
Box plot with quartile distribution for the variables Mayo (partial, endoscopic and complete), and C reactive protein (CRP) and calprotectin levels at baseline and follow-up time points. ^o^ represent outliers.

**Figure 6:**
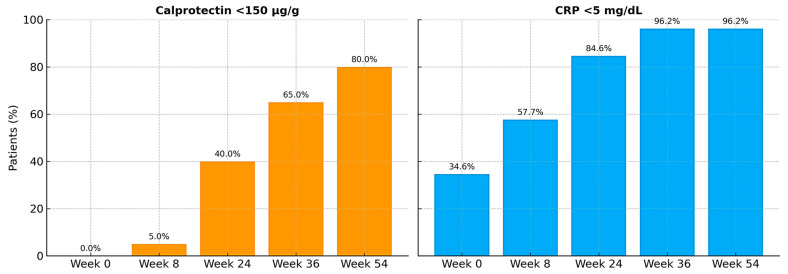
Proportion of patients meeting prespecified criteria for the variables calprotectin and C reactive protein (CRP) levels at baseline and follow-up time points.

Table 4 and Figure 5 and Figure 6 present the comparative analysis of the repeated clinical and biochemical measurements at different time points after golimumab initiation. Only patients who presented all measurements during the follow-up period were considered, therefore, the sample size is different from the total and by variable.

### 3.4. Predictive Factors of Clinical Response and Remission

At week 24, Montreal E3 extension (extensive colitis) was negatively associated with clinical response (HR = 0.15; 95% CI: 0.03–0.86; *p* = 0.033) and remission (HR = 0.08; 95% CI: 0.01–0.65; *p* = 0.018). The concomitant use of azathioprine was associated with a higher rate of clinical response at week 24 (HR = 11.97; 95% CI: 2.14–66.89; *p* = 0.005). Variables such as age, sex, disease duration, smoking, prior use of corticosteroids and biologics did not have a significant impact on the analyzed outcomes. The results of these analyses are presented in Table 5.

### 3.5. Requirement for Golimumab Dose Optimizations

**Figure 7:**
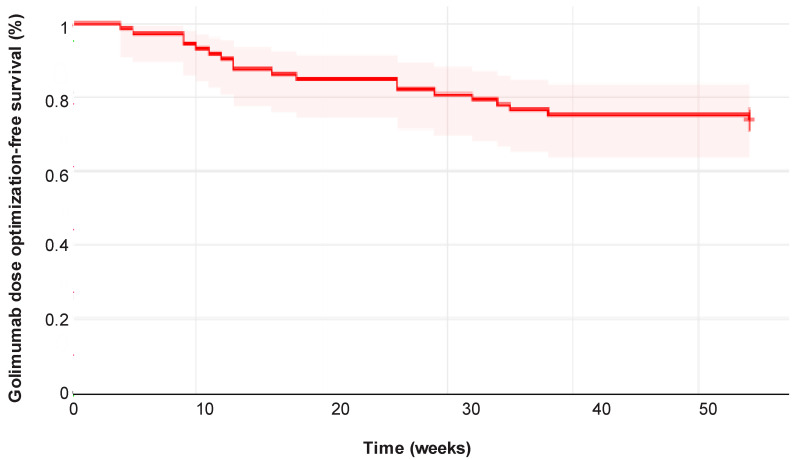
Cumulative golimumab dose optimization-free survival in the study cohort. The median time to golimumab dose optimization was 26 weeks (IQR 20).

During follow-up, 21 of 73 patients (28.7%) required dose optimization of golimumab. Of these patients, 52.3% underwent early optimization and 47.6% underwent late optimization. The cumulative probability of maintaining golimumab treatment without dose optimization was 83.6% (95% CI 76 to 92) at 54 weeks (Figure 7). All optimizations were carried out by increasing the golimumab dose. In one patient dose was escalated to 200 mg eq4w. In the remaining 20 patients (with a baseline body weight of less than 80 kg) the golimumab dose was escalated to 100 mg eq4w. Golimumab dose optimization was successful in most patients, with 18 (85.7%) of those who underwent dose optimization retaining golimumab at the last follow-up.

### 3.6. Safety

Adverse events occurred in 24 patients (32.8%), including 13 women (54.2%) and 11 men (45.8%), and are summarized in Table 6. Upper respiratory tract infection occurred in four patients, and lower respiratory tract infection in one patient. Four patients had skin reactions and seven had reactions at the injection site. One patient presented with headache and one with new onset psoriasis. Adverse events leading to golimumab discontinuation occurred in three patients (4.1%). No major adverse cardiovascular events, malignancies or deaths were reported.

## 4. Discussion

This observational study reports the real-world outcomes of golimumab in a multicenter Brazilian cohort of patients with moderate-to-severe active UC. The results of the study confirmed, in a different geographical area like Brazil, the effectiveness and safety of golimumab for UC seen in prior observational studies [13,15]. Notably, after a median follow-up of almost 60 weeks, 86.1% of patients maintained clinical benefit while receiving golimumab maintenance therapy.

Our results support the efficacy of golimumab as a therapy for UC, for both induction and maintenance. Rates of clinical response at week 8 and rates of CS-free remission at weeks 24 and 54 are similar to those previously reported in other observational studies [13,14,15,16]. At week 8, 57.5% of patients had a clinical response, and almost two-thirds achieved CS-free clinical remission within 54 weeks. Furthermore, the efficacy of golimumab as a therapy for UC was supported by objective evidence such as the progressive reduction in CRP and fecal calprotectin levels observed at successive time points after initiation of golimumab. In addition, over 80% of patients achieved endoscopic remission at weeks 24 and 54 (among patients who underwent endoscopic assessment). Only 13.7% of patients discontinued golimumab during follow-up, with the median time to golimumab discontinuation being 25.3 weeks.

Predictors such as age, sex, disease duration, smoking, and use of corticosteroids did not have a significant impact on the analyzed outcomes. Although prior exposure to biologic therapies has been negatively associated with treatment outcomes in several studies, our findings did not replicate this association, as previous use of biologics showed no significant impact on the analyzed outcomes [12,15,21]. However, greater disease extension was associated with a negative impact on achieving clinical response and remission at week 24. Additionally, the concomitant use of immunomodulators was positively associated with clinical outcomes at week 24, consistent with findings from other real-world studies demonstrating that the combination of golimumab and immunosuppressants can enhance treatment response [14]. Despite these findings, the model demonstrated limited explanatory power, as reflected by the low R-squared values, while the lack of global significance (*p* > 0.05) underscores the need for caution in interpreting the data. It is possible that unaccounted variables or unassessed interactions play a relevant role in predicting the outcomes.

In observational studies, outcomes with biologics have often been better than expected based on the results of RCTs. This may be the result of the clinicians’ greater freedom to dose-escalate biologics or to use concomitant treatments, like topical therapy, that are prohibited in trials. Previous studies using infliximab or adalimumab in UC demonstrated that dose optimization is often necessary to maintain a durable response [22,23,24,25,26,27]. Observational studies reported that optimizing golimumab dosing is necessary to manage suboptimal clinical response or loss of response in over 20% of patients [13,14,15,28,29,30]. In our study, 28.7% of patients required golimumab dose optimization, with the median golimumab dose optimization-free survival being 26 weeks. The dose optimization method was homogeneous, always by increasing golimumab dose without change in the eq4w interval. Golimumab dose optimization was clearly beneficial, with over 85% of patients recovering response after dose escalation and avoiding golimumab discontinuation at last follow-up. The persistence with golimumab seen in this study is remarkable and we believe it is related to the high short and long-term efficacy, which can be further improved by rescuing patients with secondary loss of response through dose escalation. The colectomy rate in our cohort was low, with only four patients (5.47%) needing colectomy during follow-up, three of them being treatment-refractory biologic-experienced patients. This result is consistent with observational studies evaluating colectomy rates with golimumab in treatment-refractory patients with moderate-to-severe active UC [15,16]. Once again, we believe that the possibility of free dose escalation in our study may be associated with the low reported colectomy rate. A prior study showed that secondary non-responders who discontinued golimumab without attempting dose optimization had the worst results regarding the need for colectomy [15]. Four-year follow-up data from the PURSUIT Maintenance Extension show a reduced rate of colectomies after the first year, suggesting potential favorable long-term outcomes in real-world studies [31,32].

The safety profile of golimumab was consistent with other observational studies, with less than 5% of patients discontinuing treatment due to adverse events [14,15,16,21,33]. No new safety concerns with the use of golimumab emerged during the entire follow-up period.

The main limitation of our study was the retrospective design, as there were patients with missing data on efficacy at key time points. Endoscopies could not be prespecified and were limited to those performed in routine clinical practice. Another limitation was that decisions regarding golimumab optimization, withdrawal or CS reduction were not standardized and were left to the discretion of the attending gastroenterologist. Dose optimization was performed based solely on clinical criteria, as golimumab serum levels and anti-drug antibodies measurement was not available in any of the centers participating in the study. Drug levels and antidrug antibodies are highly relevant to our understanding of the mechanisms of the primary or secondary loss of response to golimumab and can help guide therapeutic decisions [34,35].

The real-world evidence we have on golimumab for UC comes almost entirely from studies conducted in Europe, with a few isolated studies from North America [11,12,13,14,15,21,28,29,33,34]. Real-world studies confirming the efficacy of golimumab in different populations and countries are therefore of great interest. The classification of ethnicity in Brazil differs from that used in the United States or Europe, where all Brazilians are often categorized as “Latino.” In contrast, the Brazilian Institute of Geography and Statistics (IBGE) recognizes “Pardos” (brown) as a distinct category representing individuals of mixed ancestry (European, African, and sometimes Indigenous), which is highly prevalent in Brazil. These data are the first reported in a geographic area with a similar ethnic composition, thus providing novel insights into this population. As this is the first observational study on outcomes of golimumab for UC in Brazilian patients, mirroring real-world clinical practice, we believe it has the potential to provide valuable information for clinicians involved in the treatment of UC in diverse geographic areas. A major strength of our study is that it represents the first real-world data on golimumab in a population with a distinct ethnic distribution, reflecting the heterogeneous ancestry of Brazil and thereby adding valuable perspectives to the worldwide evidence base.

## 5. Conclusions

Our real-world experience in the treatment of moderate-to-severe UC with golimumab supports its efficacy and safety as an induction and maintenance therapy in both biologic-naive and biologic-experienced patients, leading to a high persistence rate with golimumab maintenance. Optimization of golimumab was clearly beneficial, with most patients maintaining golimumab therapy after dose escalation. Based on these results, golimumab appears to be a potential treatment option for moderate-to-severe UC in Brazil. However, further research involving larger patient groups and longer-term follow-up is required to confirm these findings.

## Figures and Tables

**Table 1:** Mean, standard deviation (SD), coefficient of variation (CV%), and quartile distribution of the quantitative variables that characterize the sample (*n* = 73).

	Mean	SD	CV%		Quartile	
				Median	25th	75th
Age (years)	40.7	15.3	37.6	38.0	29.0	52.0
Age at diagnosis (years)	34.0	13.9	40.7	32.0	24.0	48.0
Baseline weight (kg)	66.2	13.2	19.9	65.0	59.0	79.0
Baseline height (m)	1.67	0.09	5.23	1.66	1.60	1.72
Baseline BMI (kg/m^2^)	27.4	25.1	91.6	23.3	21.7	26.7

First quartile (25th); third quartile (75th).

**Table 2:** Demographic and clinical characteristics at baseline. Absolute (*n*) and relative frequency distribution of qualitative variables for sample characterization (*n* = 73).

		*n*	% Total	% Valid
Sex	Male	45	61.6	61.6
	Female	28	38.4	38.4
Ethnicity	White	49	67.1	72.1
	Black	11	15.1	16.2
	Brown	8	11.0	11.8
	No data	5	6.8	
Smoking	No	60	82.2	84.5
	Yes	11	15.1	15.5
	No data	2	2.7	
Comorbidity	No	43	58.9	59.7
	Yes	29	39.7	40.3
	No data	1	1.4	
Extraintestinal manifestations (EIM)	No	39	53.4	54.2
	Yes	33	45.2	45.8
	Total	72	98.6	100.0
	No data	1	1.4	
Extent of disease (Montreal)	E1	14	19.2	21.5
	E2	28	38.4	43.1
	E3	23	31.5	35.4
	No data	8	11.0	
Corticosteroid	No	7	9.6	10.0
	Yes	63	86.3	90.0
	No data	3	4.1	
5-Aminosalicylates	No	29	39.7	39.7
	Yes	44	60.3	60.3
Immunomodulators	No	67	91.8	91.8
	Yes	6	8.2	8.2
Infliximab (IFX)	No	64	87.7	87.7
	Yes	9	12.3	12.3
Adalimumab (ADA)	No	68	93.2	93.2
	Yes	5	6.8	6.8
Vedolizumab (VEDO)	No	60	82.2	82.2
	Yes	13	17.8	17.8
Ustekinumab (UST)	No	72	98.6	98.6
	Yes	1	1.4	1.4
Certolizumab pegol (CTZ)	No	70	95.9	95.9
	Yes	3	4.1	4.1

**Table 3:** Mean, standard deviation (SD), coefficient of variation (CV%), and quartile distribution of Mayo (partial, endoscopic, and complete), CRP, and Calprotectin at baseline (*n* = 73).

Variable	*n*	Mean	SD	CV%	Quartile		
					Median	25th	75th
Partial Mayo score (0–9 points)	63	6.53	1.85	28.3	7.00	5.00	8.00
Mayo endoscopic subscore (0–3 points)	72	2.47	0.71	28.8	3.00	2.00	3.00
Mayo score (0–12 points)	63	8.39	2.54	30.3	9.00	8.00	10.00
CRP (mg/dL)	66	11.10	12.70	114.4	6.85	2.06	12.44
Calprotectin (mcg/g)	62	1939.7	1375.6	70.9	1800.0	848.5	2925.0

First quartile (25th); third quartile (75th).

**Table 4:** Comparison of the median and interquartile range of partial Mayo score, complete Mayo score, endoscopic Mayo subscore, and C reactive protein (CRP) and calprotectin levels between baseline and the different time points after golimumab initiation.

	Median	25th	75th	*p*-Value
Partial Mayo Week 0 (0–9 points)	7.00 ^a^	5.00	8.00	<0.001 *
Partial Mayo Week 8 (0–9 points)	3.00 ^b^	2.50	6.00	
Partial Mayo Week 24 (0–9 points)	2.00 ^c^	1.00	4.00	
Partial Mayo Week 36 (0–9 points)	1.00 ^d^	0.00	2.00	
Partial Mayo Week 54 (0–9 points)	1.00 ^d^	0.00	2.00	
Complete Mayo Week 0 (0–12 points)	9.00 ^a^	8.00	10.00	<0.001 *
Complete Mayo Week 24 (0–12 points)	4.00 ^b^	2.00	5.00	
Complete Mayo Week 54 (0–12 points)	2.00 ^c^	0.00	3.00	
Endoscopic Mayo Week 0 (0–3 points)	3.00 ^a^	2.00	3.00	<0.001 *
Endoscopic Mayo Week 24 (0–3 points)	1.00 ^b^	0.00	2.00	
Endoscopic Mayo Week 54 (0–3 points)	0.00 ^c^	0.00	1.00	
CRP week 0 (mg/dL)	6.85 ^a^	2.06	12.44	<0.001 *
CRP week 8 (mg/dL)	3.45 ^b^	1.05	6.80	
CRP week 24 (mg/dL)	2.45 ^c^	0.90	4.00	
CRP week 36 (mg/dL)	2.10 ^c,d^	0.40	3.50	
CRP week 54 (mg/dL)	1.06 ^d^	0.35	2.00	
Calprotectin week 0 (mcg/g)	1800.0 ^a^	848.5	2925.0	<0.001 *
Calprotectin week 8 (mcg/g)	569.0 ^b^	246.0	1362.8	
Calprotectin week 24 (mcg/g)	220.0 ^c^	80.8	632.0	
Calprotectin week 36 (mcg/g)	128.0 ^c^	76.5	297.5	
Calprotectin week 54 (mcg/g)	63.0 ^c^	39.0	121.5	

First quartile (25th); third quartile (75th). * indicates significant difference between weeks by Friedman test for *p*-value ≤ 0.050. For median, different letters (a, b, c, d) indicate a significant difference using the Holm–Sidak post hoc test for *p*-value ≤ 0.050.

**Table 5:** Multivariable Cox regression analysis of factors associated with treatment response and remission at Weeks 24 and 54.

Exposure Factor and Predictors	Level	Dependent Variable HR (Multivariable)			
		Response Week 24	Remission Week 24	Response Week 54	Remission Week 54
Sex	Male	-	-	-	-
	Female	HR = 1.02 95% CI: 0.37–2.84 *p* = 0.968	HR = 1.31 95% CI: 0.45–3.78 *p* = 0.616	HR = 1.45 95% CI: 0.62–3.41 *p* = 0.397	HR = 1.59 95% CIO: 0.66–3.84 *p* = 0.305
Smoking	No	-	-	-	-
	Yes	HR = 0.18 95% CI: 0.02–1.36 *p* = 0.097	HR = 0.21 95% CI: 0.02–2.64 *p* = 0.229	HR = 0.85 95% CI: 0.27–2.68 *p* = 0.787	HR = 0.79 95% CI: 0.22–2.80 *p* = 0.712
Montreal Classification (disease extension)	E3	HR = 0.15 95% CI: 0.03–0.86 *p* = 0.033 *	HR = 0.08 95% CI: 0.01–0.65 *p* = 0.018 *	HR = 0.27 95% CI: 0.07–1.09 *p* = 0.067	HR = 0.26 95% CI: 0.06–1.15 *p* = 0.076
Corticosteroid at baseline	No	-	-	-	-
	Yes	HR = 0.57 95% CI: 0.11–3.02 *p* = 0.508	HR = 0.50 95% CI: 0.08–2.94 *p* = 0.441	HR = 0.78 95% CI: 0.19–3.13 *p* = 0.724	HR = 0.59 95% CI: 0.15–2.35 *p* = 0.450
Concomitant immunomodulators	No	-	-	-	-
	Yes	HR = 11.97 95% CI: 2.14–66.89 *p* = 0.005 *	0.00 (0.00-Inf, *p* = 0.998)	HR = 5.31 95% CI: 0.46–60.96 *p* = 0.180)	HR = 0.00 95% CI: 0.00-Inf *p* = 0.998
Biologic exposure	No	-	-	-	-
	Yes	HR = 1.04 95% CI: 0.40–2.72 *p* = 0.940	HR = 0.82 95% CI: 0.26–2.57 *p* = 0.732	HR = 1.35 95% CI: 0.65–2.81 *p* = 0.427	HR = 1.38 95% CI: 0.64–3.01 *p* = 0.414
Age (years)	Mean (SD)	HR = 0.99 95% CI: 0.95–1.02 *p* = 0.489	HR: 0.98 95% CI: 0.98 (0.94–1.02, *p* = 0.399	HR = 0.97 95% CI: 0.94–1.01 *p* = 0.100	HR = 0.97 95% CI: 0.94–1.01 *p* = 0.120
Duration of disease (years)	Mean (SD)	HR: 1.03 95% CI: 0.94–1.13 *p* = 0.499)	HR: 1.07 95% CI: 0.97–1.17 *p* = 0.186	HR = 1.01 95% CI: 0.94–1.09 *p* = 0.814	HR: 1.02 95% CI: 0.94–1.10 *p* = 0.686
Model Metrics (Likelihood ratio test)	R-squared	0.217	0.231	0.257	0.275
	*p*-value	0.240	0.188	0.275	0.213

Note: * indicates significant effect of the independent variable within the multivariate model for *p*-value ≤ 0.05. Multivariable Cox regression analysis of clinical predictors of treatment response and remission at weeks 24 and 54. HR: adjusted hazard ratio; CI: confidence interval. Response and remission were defined according to standardized clinical criteria assessed at each time point.

**Table 6:** Adverse events reported with golimumab treatment. Data are expressed as absolute number in each adverse event (*n*) and absolute number (%) in each type of adverse event.

Type of Adverse Event	*n* (%)
Infections	9 (13.3%)
Respiratory: mild respiratory infections (5), tuberculosis (1), pneumonia (1)	
Urinary tract infections (2)	
Dermatologic	12 (16.4%)
Rash at the injection site (7), folliculitis (1), pruritus and urticaria (3), psoriasis (1)	
Neurological	1 (1.4%)
Headaches (1)	
Articular	2 (2.8%)
Arthralgia (2)	
Total	24 (32.8%)

## Data Availability

The original contributions presented in this study are included in the article. Further inquiries can be directed to the corresponding author.

## Abbreviations

The following abbreviations are used in this manuscript:

UC	Ulcerative Colitis
CS	Corticosteroid
PMS	Partial Mayo Score
5-ASA	5-Aminosalicylates
JAK	Janus Kinase
TNF	Tumor Necrosis Factor
SC	Subcutaneous
RCT	Randomized Clinical Trials
CEP	Ethics Committee
STROBE	STrengthening the Reporting of OBservational studies in Epidemiology
ANVISA	Brazilian Health Regulatory Agency
BMI	Body Mass Index
EIM	Extraintestinal Manifestations
CRP	C-reactive Protein
FC	Fecal Calprotectin
MES	Mayo Endoscopic Subscore
SD	Standard Deviation
IQR	Interquartile Range
CI	Confidence Interval
RR	Relative Risk
CV	Coefficient of Variation
IFX	Infliximab
ADA	Adalimumab
VEDO	Vedolizumab
UST	Ustekinumab
CTZ	Certolizumab pegol
mg	milligram
dL	deciliter
µg	microgram
g	gram
HR	Hazard Ratio
